# Dietary intake and biomarker status of folate in Swedish adults

**DOI:** 10.1007/s00394-016-1328-4

**Published:** 2016-10-27

**Authors:** Veronica Öhrvik, Eva Warensjö Lemming, Cecilia Nälsén, Wulf Becker, Peter Ridefelt, Anna Karin Lindroos

**Affiliations:** 1 Science Department, National Food Agency, Box 622, 751 26 Uppsala, Sweden; 2 Department of Medical Sciences, Uppsala University, Box 256, 751 05 Uppsala, Sweden

**Keywords:** Folate intake, Folate status, Red blood cell folate concentrations, Riksmaten adults 2010–11, Food intake, Swedish national dietary survey

## Abstract

**Purpose:**

National data on folate status are missing in Sweden, and regional data indicate folate insufficiency in up to more than 25% of the study populations. The objectives were to determine folate intake and status in the adult Swedish population as well as identifying dietary patterns associated with beneficial folate status.

**Methods:**

Folate intake was estimated using a web-based 4-d food record in adults aged 18–80 years (*n* = 1797). Folate status was measured as erythrocyte (*n* = 282) and plasma folate concentrations (*n* = 294). Factor analysis was used to derive a dietary pattern associated with a higher folate status.

**Results:**

Median folate intake was 246 µg/day (*Q*_1_ = 196, *Q*_3_ = 304, *n* = 1797) and for women of reproductive age 227 µg/day (*Q*_1_ = 181, *Q*_3_ = 282, *n* = 450). As dietary folate equivalents (DFE), median intake was 257 µg/day (*Q*_1_ = 201, *Q*_3_ = 323) and for women of reproductive age 239 µg/day (*Q*_1_ = 185, *Q*_3_ = 300). Low blood folate concentrations were found in 2% (erythrocyte concentrations <317 nmol/L) and 4% (plasma concentrations <6.8 nmol/L) of the participants, respectively. None of the women of reproductive age had erythrocyte folate concentrations associated with the lowest risk of neural tube defects. Dietary patterns associated with higher folate status were rich in vegetables, pulses and roots as well as cheese and alcoholic beverages, and low in meat.

**Conclusions:**

Prevalence of low erythrocyte folate concentrations was low in this population, and estimated dietary intakes are well above average requirement. However, to obtain a folate status optimal for prevention of neural tube defects major dietary changes are required and folic acid supplements recommended prior to conception.

## Introduction

Folate deficiency results in anaemia, and an optimal folate status is known to reduce prevalence of neural tube defects (NTD) [1, 2]. To maintain blood folate concentrations above cut-offs (317 nmol/L for erythrocyte folate and 6.8 nmol/L for serum folate), average requirement (AR) for folate is set to 200 µg/day and recommended intake (RI) to 300 µg/day in the Nordic Nutrition Recommendations [3]. Because of the preventive effect against NTDs of an adequate supply of folate during early pregnancy, RI for women in reproductive age is 400 µg/day [3].

According to previous Swedish national dietary surveys and cohort studies, folate intakes are in line with AR, but only a small proportion of the intakes is above RI [4–8]. This is particularly true for women of reproductive age [6, 7, 9]. However, to assess folate intake is difficult, not only as a result of the traditional limitations with dietary surveys but also because folate quantification in foods is challenging. Different analytical methods are known to cause up to 30% differences in results [10, 11]. Hence, folate intakes preferably are complemented with biomarkers for folate status and appropriate methods to assess supplement intake [12]. Regional data indicate low plasma folate concentrations in Sweden compared to other European countries [13–16]. For example, in a subsample of the Northern Sweden Health and Disease Study (*n* = 2617, age 25–74 y) fasting median plasma folate concentrations were 9 nmol/L (*Q*_1_ = 6, *Q*_3_ = 12) in women and 8 nmol/L (*Q*_1_ = 6, *Q*_3_ = 11) in men and about 30% had low blood folate concentrations defined as concentrations below 6.8 nmol/L [8].

The European Food Safety Authority (EFSA) considers erythrocyte folate concentrations the most reliable measure of folate status [17]. However, in Sweden published data on erythrocyte folate concentrations are scarce and nationally representative data on erythrocyte folate concentrations as well as plasma folate concentrations are missing. The aim of this study was to assess folate intake and folate status in the Swedish population, and to identify food patterns associated with beneficial folate status.

## Subjects and methods

### Study design and population

**Fig. 1:**
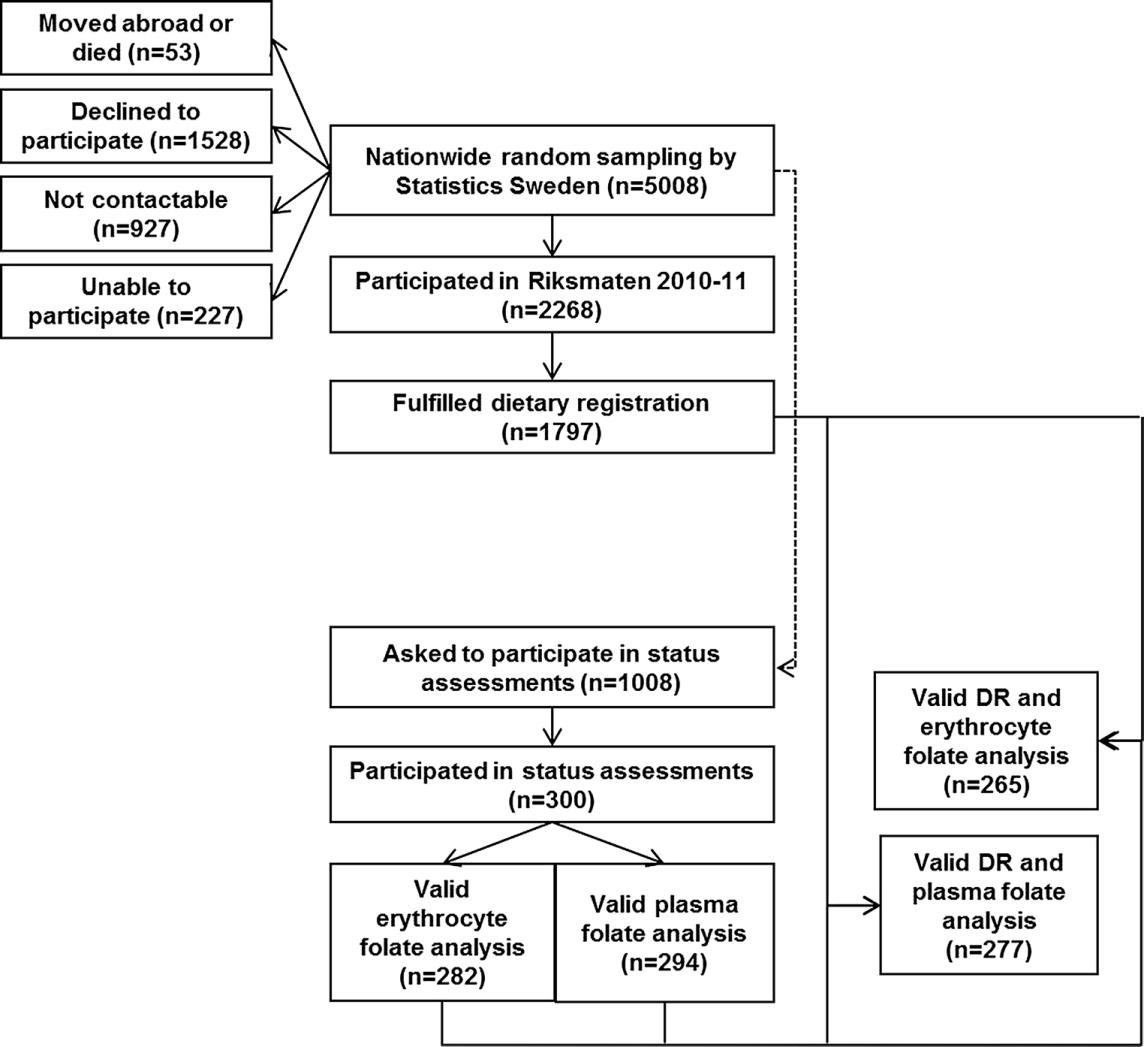
Flowchart study outline. *DR* dietary registration

Riksmaten Adults 2010–11 [18] is the most recent national dietary survey in Sweden and was conducted by the Swedish National Food Agency (NFA). The method has been validated [19, 20]. Statistics Sweden invited a representative national sample of 5008 adults aged 18–80 years to participate in the dietary survey and a subsample of 1008 of these to also participate in assessments of nutritional status (Fig. 1). The subsample was divided into seven regions according to affiliation to Swedish Occupational and Environmental Medicine Centers (OEMCs). Each region included the regional capital (Linköping, Lund, Stockholm, Umeå, Uppsala, Gothenburg and Örebro) together with two randomly selected counties in each region. An equal number of individuals were selected in each region independent of population size. Recruitment took place at four different occasions during the year to cover different seasons. Twelve individuals per part of the region and occasion were invited to participate (7 regions, 3 sites in each, the capital and two counties, four occasions and 12 individuals per round). The participation rate was 36% for the dietary survey and 30% for the subsample (Fig. 1). The study protocol was approved by the Regional Ethical Review Board in Uppsala.

### Dietary assessment

The participants reported everything they ate and drank for four consecutive days, using a web-based food diary developed by NFA [21]. Food intake and status in all seasons and all days of the week were captured by carrying out the study from May 2010 to July 2011 and by randomly assigning starting days to participants.

To estimate food and folate intake, a food composition database consisting of 1909 food items was used. Intakes of foods and beverages (gram per day) were estimated using a portion guide, household measures, numbers of portions (cups, pieces, slices) and grams. The portion guide, available as a printed booklet and in the web tool, included 24 different food categories with four to eight different reference sizes in each category. For about 30% of the foods, folate content was analysed using an accredited trienzyme microbiological method (*L. rhamnosus*, Culture Collection of the University of Gothenburg, CCUG 21452, equivalent to *L. casei* American Type Culture Collection, ATCC 7469). For about 20% of the foods, folate values were imputed from similar foods or borrowed from other national food databases, companies or scientific literature. The values for the remaining foods were calculated from recipes using analytical data on folate in ingredients and factors for losses and gains of water and fat as well as losses of folate due to heat treatment [22].

Information on supplement use and frequency was collected using a questionnaire as recommended by Bates et al. [12]. As most available folic acid supplements in Sweden contain 500 µg of folic acid (the RI for pregnant and nursing women [3]), the doses of all registered folic acid supplements were set to 500 µg of folic acid for estimation of intakes from supplements. Foods included in the survey were controlled for voluntary fortification and if relevant also fortification level. Dietary folate equivalents (DFE) were calculated according to the formulae: 1 μg DFE = 1.0 μg food folate = 0.6 μg folic acid added to foods = 0.5 μg folic acid taken without food [23].

The Goldberg cut-off (Black), which defines the probability of adequately reported energy intake, based on body size and physical activity level, was used to identify low-energy reporters. Information on physical activity level (PAL) at work and leisure time was collected as part of the dietary record on a four-grade scale. Criteria for acceptable energy intake were a quota of energy expenditure to basal metabolic rate within the confidence interval (0.93–3.01).

### Blood sampling and analysis

Non-fasting blood samples for folate status were collected in coded sterile Vacutainer™ tubes from BD (Belliver Industrial Estate, Plymouth, UK) by nurses at the OEMCs. Samples were kept at −20 °C until analysis.

Whole blood samples for erythrocyte folate status were collected in 3.0-mL EDTA tubes, and analysed using a chemiluminescence immunoassay method at the Karolinska University Hospital, Sweden. Erythrocytes were hemolysed using ‘RBC folate Hemolyzing Reagent’ (Roche Diagnostics GmbH, Mannheim, Germany) and quantified as serum folate (range 45–1407 nmol/L, intra-assay CV 3%). Erythrocyte folate concentrations were not corrected for serum folate concentrations.

Plasma folate samples were collected in 3.5-mL PST tubes and analysed at the Clinical Chemistry and Pharmacology Department at Uppsala University Hospital, Sweden. Plasma folate (cat. no. 1P74-35, Abbott Laboratories, Abbott Park, IL, USA) was analysed by a chemiluminescence immunoassay method on an Abbott Architect ci8200 analyser. The laboratory was accredited according to SS-EN ISO/IEC 15189:2007. As part of the accreditation procedures, the laboratory participated in interlaboratory external proficiency testing schemes for P-folate from EQUALIS AB, Uppsala and Labquality OY, Helsinki. The total analytical imprecision of plasma folate measurements was 12 and 7 CV % at 4 and 35 nmol/L, respectively. Cut-off level was 8 nmol/L.

### Statistical analysis

Data were expressed as median, lower quartile (*Q*_1_) and upper quartile (*Q*_3_) or as percentage. Folate intakes were presented including and excluding low-energy reporters. Erythrocyte and plasma folate concentrations were not normally distributed as tested using the Shapiro–Wilk test, so nonparametric methods were used. The Wilcoxon–Mann–Whitney rank sum test was used to test if sex, age, income, fruit and vegetable consumption or low-energy reporting affected intake and status. The Kruskal–Wallis test was used to compare if education level affected intake and status. Associations (folate intake and biochemical parameters) were assessed by Spearman’s ranked correlation coefficients.

Food patterns associated with folate status parameters were identified using factor analysis followed by robust regression analysis. Food groups (in gram per day) included in the factor analysis were: vegetables, pulses and roots; fruit and berries; potatoes; bread; rice and grains; pasta; porridge and gruels; breakfast cereals; meat including offal and blood products; poultry; sausages; fish and shellfish; egg; milk, fermented milk and yoghurt; cheese; spreads and butter; coffee, tea and water; fruit and vegetable juice; soft drinks, sports and energy drinks; beer, wine and spirits (alcoholic beverages); jam, marmalade and apple sauce; ice cream; candy and chocolate; buns, biscuits and cakes; sugar, syrup, honey and artificial sweeteners; pizza, pie and pirogue; pancakes, waffles and crepes; soup; sauces; and diet and nutritional supplements, i.e. bars, powder. Rotated factor loadings above 0.300 were included in the final analysis. To simplify the interpretation of the selected factors, the varimax (orthogonal) rotation was applied, this has as it is rational the provision of uncorrelated factors with a few large loadings and as many loadings as possible close to zero.

STATA version 12.1 (STATA Corp.) was used for all analyses. A two-sided *p* < 0.05 was regarded as statistically significant.

## Results

### Basic demographic characteristics of participants are presented in Table 1

#### Folate intake

Folate median intake in the total population (*n* = 1797) was 250 µg/day, corresponding to 300 µg/10 MJ (Table 2). Excluding of low-energy reporters resulted in significantly higher median folate intake, about 260 µg/day (Table 2). Women of reproductive age (18–44 y) had a lower intake (227 µg/day) than women aged 45–80 y (245 µg/day) (*p* = 0.001), and 44% had an intake below average requirement (AR, 200 µg/day, [3]). Only 5% reported an intake above 400 µg/day (RI for women in reproductive age [3]) (Table 2).

**Table 1:** Demographic description of participants

	Folate intake	Folate status
*N*	1797	300
Women (%)	56	54
Age (years)	49 (35–62)	50 (37–64)
BMI (kg/m^2^)	24.7 (22.5–27.6)	24.7 (22.6–27.5)
PAL	1.7 (1.6–1.8)	1.7 (1.6–1.8)
Vegetable intake (g/d)	163 (109–224)	186 (124–241)
Fruit and berries intake (g/d)	109 (38–191)	113 (44–202)
Intake of folic acid supplements number (%)	20 (1)	20 (8)

Values are median (*Q*_1_–*Q*_3_) unless otherwise stated

*PAL* physical activity level

**Table 2:** Folate intake by sociodemographic variables and fruit and vegetable intake

	Folate intake (µg/d)					Folate intake (µg/10 MJ)					DFE (µg/day)	
	All		Low-energy reporters excluded			All		Low-energy reporters excluded			All	
	*n*	Median (*Q*_1_–*Q*_3_)	*n*	Median (*Q*_1_–*Q*_3_)	*p*	*n*	Median (*Q*_1_–*Q*_3_)	*n*	Median (*Q*_1_–*Q*_3_)	*p*	*n*	Median (*Q*_1_–*Q*_3_)
All	1797	246 (196–304)	1467	261 (215–318)	0.000	1797	302 (253–367)	1467	298 (250–360)	0.210	1797	257 (201–323)
Male	792	257 (204–316)	627	276 (229–332)	0.000	792	278 (232–336)	627	273 (230–327)	0.190	792	268 (210–336)
Female	1005	237 (189–294)	840	249 (207–307)	0.000	1005	326 (272–390)	840	323 (271–383)	0.360	1005	247 (195–211)
Female aged 18–44 y	450	227 (181–282)	369	240 (199–294)	0.002	450	301 (256–355)	369	296 (254–345)	0.400	450	239 (185–300)
		*p* (sex) < 0.001		*p* (sex) < 0.001			*p* (sex) < 0.001		*p* (sex) < 0.001			*p* (sex) < 0.001
		*p* (female) < 0.001		*p* (female) = 0.001			*p* (female) < 0.001		*p* (female) < 0.001			*p* (female) < 0.001
*Fruit and vegetable consumption*												
Fruit and vegetables <200 g/d	519	191 (152–235)	354	211 (171–251)	0.000	519	249 (213–293)	354	236 (205–276)	0.002	519	198 (155–248)
Fruit and vegetables 200–350 g/d	680	239 (201–284)	569	250 (211–290)	0.004	680	299 (261–347)	569	294 (258–337)	0.110	680	249 (206–298)
Fruit and vegetables 350–500 g/d	376	286 (242–328)	331	292 (249–331)	0.100	376	340 (287–395)	331	333 (283–381)	0.170	376	294 (248–344)
Fruit and vegetables >500 g/d	222	354 (304–412)	213	355 (306–414)	0.730	222	401 (347–471)	213	396 (344–465)	0.640	222	355 (309–435)
		*p* < 0.001		*p* < 0.001			*p* < 0.001		*p* < 0.001			*p* < 0.001
*Income*												
Income < median	840	237 (186–294)	682	250 (209–307)	0.000	840	303 (254–373)	682	301 (250–367)	0.450	840	248 (194–315)
Income ≥ median	957	255 (202–312)	785	269 (221–324)	0.000	957	300 (252–362)	785	295 (250–355)	0.290	957	265 (206–330)
		*p* < 0.001		*p* < 0.001			*p* = 0.420		*p* = 0.300			*p* < 0.001
*Level of education*												
Elementary school	214	230 (183–296)	157	250 (200–313)	0.007	214	305 (262–371)	157	292 (252–351)	0.190	214	243 (187–312)
High school	712	233 (190–286)	573	249 (208–295)	0.000	712	289 (244–349)	573	288 (240–344)	0.380	712	243 (196–299)
College or university	755	267 (214–326)	663	277 (230–333)	0.011	755	318 (263–381)	663	316 (263–377)	0.590	755	280 (221–344)
		*p* < 0.001		*p* < 0.001			*p* < 0.001		*p* < 0.001			*p* < 0.001

The Wilcoxon–Mann–Whitney rank sum test was used to test if sex, age, income, fruit and vegetable consumption or low-energy reporting affected intake. The Kruskal–Wallis test was used to compare if education level affected intake

*DFE* Dietary Folate Equivalents

Among those reporting a fruit and vegetable intake of at least 500 grams, more than 75% had an intake above RI for adults (300 µg/day [3]). Higher income and higher education were also associated with a higher folate intake (Table 2). Men reported a higher folate intake in µg/day. However, after energy adjustment men reported a significantly lower folate intake than women (Table 2).

Folic acid fortification is voluntary in Sweden, but the number of fortified products is limited and consumption of folic acid supplements is low (about 1%, Table 1). Thereby estimation of DFE intakes resulted in a bimodal distribution with consumers of fortified food or supplements separated from non-consumers. Estimated median DFE intake was 257 µg/day (*Q*_1_ = 201, *Q*_3_ = 322) compared to food folate intake 246 µg/day. Also for women of reproductive age, DFE intake was higher, 239 µg/day, than food folate intake, 227 µg/day.

#### Folate status

Median erythrocyte folate concentration was 460 nmol/L and plasma folate concentration 14 nmol/L (Table 3). Men had significantly higher erythrocyte folate concentrations but lower plasma concentrations (Table 3). Excluding consumers taking multivitamins (*n* = 24) did not alter the results for erythrocytes (460 nmol/L, *Q*_1_ = 410, *Q*_3_ = 520), but removed the gender difference for plasma folate concentrations. Despite significantly different folate intakes, there was no difference in folate status between women aged 18–44 and 45–80 y, between different income or education levels. However, a higher fruit and vegetable intake was associated with a significantly higher folate status (Table 3).

**Table 3:** Erythrocyte and plasma folate concentrations by sociodemographic variables and fruit and vegetable intake

	Erythrocyte folate (nmol/L)				Plasma folate (nmol/L)			
	*n*	Median	*Q*_1_	*Q*_3_	*n*	Median	*Q*_1_	*Q*_3_
All	282	460	419	540	294	14	10	19
Male^a^	124	480	424	559	127	13	10	18
Female^a^	141	450	390	500	150	15	11	19
Female aged 18–44 y	61	440	380	481	66	14	11	18
		*p* (sex) = 0.007				*p* (sex) = 0.019		
		*p* (female) = 0.070				*p* (female) = 0.380		
*Fruit and vegetable consumption*								
Fruit and vegetables <200 g/d	59	450	400	520	63	13	9.6	16
Fruit and vegetables 200–350 g/d	104	460	400	535	110	14	10	19
Fruit and vegetables 350–500 g/d	65	450	420	569	66	14	11	18
Fruit and vegetables >500 g/d	37	490	460	670	38	16.5	13	22
		*p* = 0.016				*p* = 0.009		
*Income*								
Income < median	133	460	419	530	137	13	10	17
Income ≥ median	146	465	420	550	154	14	11	19
		*p* = 0.260				*p* = 0.210		
*Level of education*								
Elementary school	26	445	420	540	30	12	9	17
High school	117	460	419	530	122	13	10	18
College or university	119	470	423	550	122	15	12	20
		*p* = 0.600				*p* = 0.370		

The Wilcoxon–Mann–Whitney rank sum test was used to test if sex, age, income, fruit and vegetable consumption or low-energy reporting affected status. The Kruskal–Wallis test was used to compare if education level affected status

^a^Data on gender are missing for erythrocyte folate (*n* = 17) and plasma folate (*n* = 17)

Folate deficiency [3] was found in 2% (*n* = 5; erythrocyte concentrations <317 nmol/L) and 4% (*n* = 11; plasma concentrations <6.8 nmol/L) of the participants, respectively.

Only three individuals, all consuming folic acid supplements, had erythrocyte folate concentrations above 906 nmol/L, the level associated with the lowest risk for NTD [24, 25]. None of these were women of reproductive age.

**Fig. 2:**
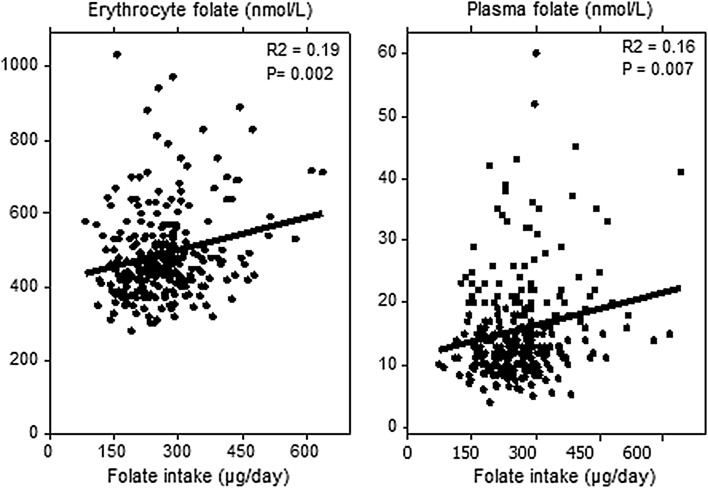
Correlation between folate intake (µg/day) and erythrocyte folate concentrations (*left*, *n* = 265) and plasma folate concentrations (*right*, *n* = 277). Consumers of supplements are excluded

Folate intake correlated with erythrocyte and plasma folate concentrations (Fig. 2).

#### Dietary pattern associated with folate status

**Fig. 3:**
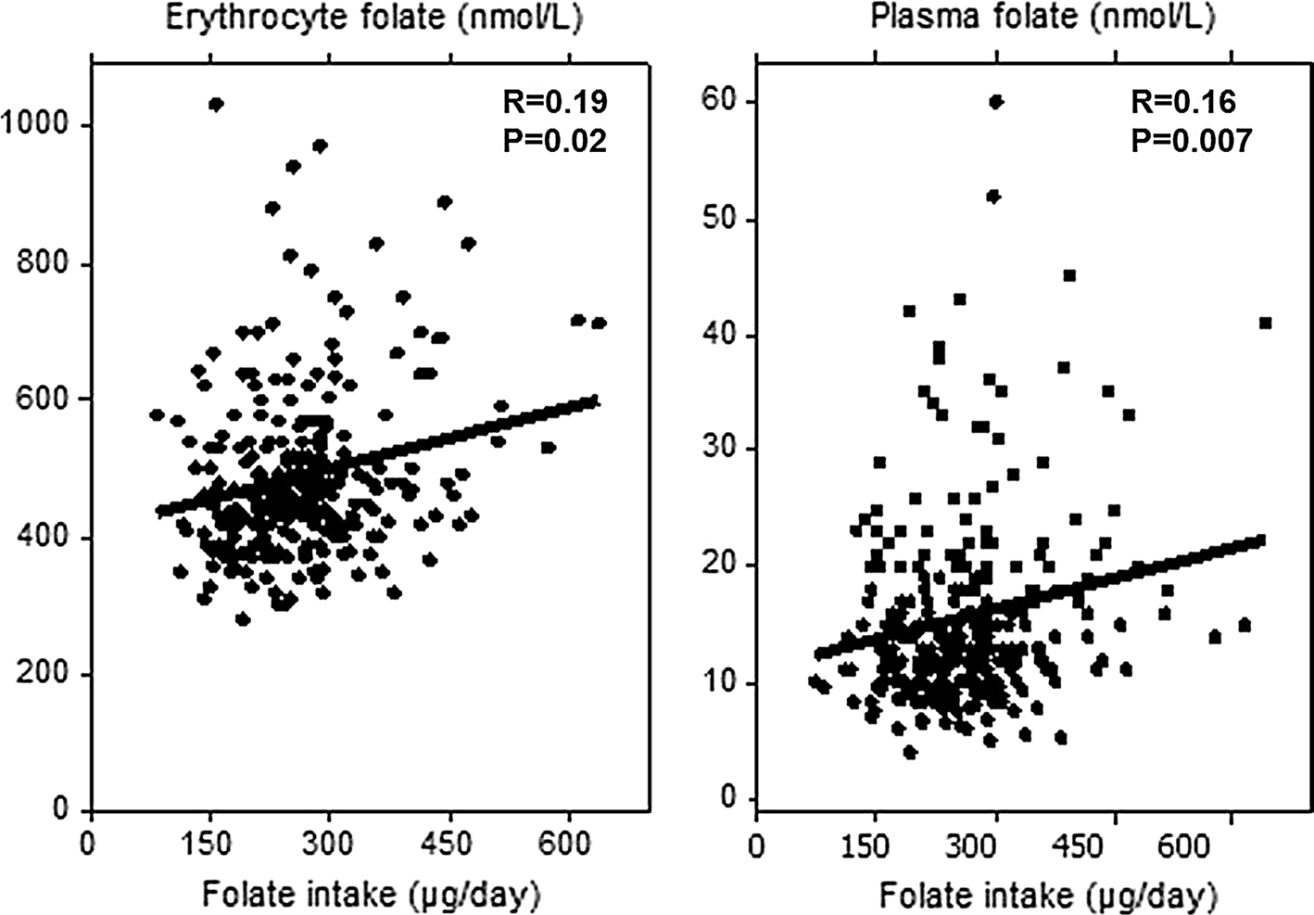
Associations between erythrocyte folate concentrations and different food groups (intake in grams per day) identified by factor analysis (factor loadings >0.300). *p < 0.05; **p < 0.01; ***p < 0.005

Total variance of erythrocyte folate concentration that was explained by the food groups identified by factor analysis was 48.4%. Consumption of alcoholic beverages, vegetables and cheese was significantly associated with a higher folate status, whereas meat consumption was significantly associated with lower folate status (Fig. 3).

## Discussion

### Folate intake

To maintain blood folate concentrations above cut-off values (plasma and erythrocyte folate concentrations below 6.8 and 317 nmol/L, respectively), NNR set average requirement (AR) to 200 µg/day and recommended intake (RI) to 300 µg/day [3]. Previous studies in Sweden report average or median folate intakes ranging from AR and up to RI (Table 4) [4–9, 26–31]. In this survey, nearly 75% reported a folate intake above AR and about 25% above RI. However, out of the 450 women aged 18–45 years in this survey, only 17 (3%) reported an intake according to RI for women of reproductive age (400 µg/day). Of these, 3 women aged 18–30 years had intakes between 400 and 410 µg/day, and the rest were aged 31–44 years. Among these one ate a fortified bar contributing with 235 µg folic acid per day, one reported an average intake (during the four days) of 23 g of reindeer liver contributing with more than 500 µg folate/day and six reported fruit and vegetables intakes between 600 and 1100 g per day. This indicates how difficult it is to meet RI for women of reproductive age from ordinary foods, although low-energy reporting of dietary intake may add uncertainty. However, estimations of folate intakes are always less certain than, e.g. estimations of more stable micronutrients due to a combination of more complex food analysis and uncertain factors for recipe calculations. Therefore, folate intakes should preferably be evaluated together with biomarker data. Moreover, the use of a web-based self-reporting method could introduce a random error as all participants may be considered as individual coders. In addition, interviewers might be able to retrieve more detailed information on the foods compared with the self-reporting method. On the other hand, using interviewers for coding might introduce systematic errors and furthermore external coding errors were minimised by using the self-assisted web-based dietary record.

**Table 4:** Summary previous studies on folate intake and status in Sweden

Study type	*n*	Year	% (male)	Age (years)	Region	Plasma folate (nmol/L)^a^	Fasting (yes/no)	Intake (µg/day)	Intake estimation method	References
*Children and youths*										
Riksmaten barn	590	2003	51	4	National	n.d.	n.a.	158 (129–193)^a^	4 day record	[9]
	889		50	8		n.d.	n.a.	186 (153–221)^a^		
	1016		51	11		n.d.	n.a.	173 (139–218)^a^		
Cohort European Youth Heart Study	194	1989–1999	0	16 ± 4^b^	Stockholm and Örebro	n.d.	n.a.	227 ± 104^b^	24-h recall	[26]
	185		100	16 ± 4^b^		n.d.	n.a.	285 ± 109^b^		
	138		0	10 ± 4^b^		n.d.	n.a.	206 ± 76^b^	24-h recall and food record	
	163		100	10 ± 4^b^		n.d.	n.a.	205 ± 88^b^		
*Pregnant*										
PregNut pregnant	176	2006–2009	0	<35	Larger Umeå area	n.d.	n.a.	277 (253–301)^c^	66 item FFQ	[27]
PregNut pregnant incl suppl	176					n.d.	n.a.	423 (386–460)^d^		
PregNut non-pregnant	103					n.d.	n.a.	300 (274–326)^c^		
PregNut non-pregnant incl suppl	103					n.d.	n.a.	353 (316–389)^c,d^		
Case–control	921	1996–1998	0	Pregnancy week 6–12	Uppsala	8.7 ± 5.2^b^	Yes	n.d.	n.a.	[28]
*Adults*										
Riksmaten	625	1997–1998	0	>17	National	n.d.	n.a.	211 (176–250)^a^	7 day record	[7]
	589		100	>17		n.d.	n.a.	223 (185–266)^a^		
HULK 7 d record	842	1989	0	15–74	National	n.d.	n.d.	187 (127–270)^e^	7 day record	[6]
	809		100	15–74		n.d.	n.d.	218 (146–321)^e^		
The Swedish Mammography Cohort	61,433	1987–1990	0	41–74	Västmanland/Uppsala county	n.d.	n.a.	234 ± 50^b^	67 item FFQ	[5]
Malmö Diet and Cancer cohort	11,307	1991–1996	0	≥50	Malmö	n.d.	n.a.	287 ± 318^f^	Diet-history method: 7 d record 168 item FFQ and interview	[4]
	408		0	62 ± 4.9		12.2 ± 8.1^g^	No	227 ± 62^g^ DFE = 307 ± 447^d,g^		[29]
Cohort *NSHDS*	1791	1985–	100	25–74	Norrbotten/Västerbotten	8.0 (5.9–10.9)^a^	Yes and no	244 (198–306)^a^	65–84 FFQ	[8]
	1055	1985–	100	>59		8.3 (6.3–11.5)^a^		n.d.	n.a.	
	729	1985–	100	≤59		7.6 (5.8–10.3)^a^		n.d.	n.a.	
	826	1985–	0	25–74		8.8 (6.3–12.2)^a^		226 (179–274)^a^	65–84 FFQ	
	497	1985–	0	>59		8.8 (6.2–13.0)^a^		n.d.	n.a.	
	329	1985–	0	≤59		8.8 (6.6–11.7)^a^		n.d.	n.a.	
Cohort Betula	961	n.d.	47	35–80	Umeå	14.4 (7.6–46.8)^h^	No	n.d.	n.a.	[30]
*Elderly*										
Cohort	161	n.d.	43	77 ± 6	Älvkarleby	8.8 ± 4.4^b^	Yes	n.d.	n.a.	[31]

Plasma folate concentrations were measured using immunoassays

*n.d.* no data, *n.a.* not applicable, *FFQ* food frequency questionnaire

^a^Median (Q_1_, Q_3_); ^b^ mean ± stddev; ^c^ mean ± 95% CI; ^d^ folic acid × 1.7/DFE; ^e^ median (P10–P90); ^f^ crude mean ± stddev; ^g^ geometric mean ± stddev; ^h^ median (P5–P95)

Despite the voluntary fortification legalisation in EU, the number of fortified products on the Swedish market is scarce [32]. Some breakfast cereals (estimated to 40% of the market), some bars (about 30%) and some beverages (about 5%) are currently fortified with folic acid in Sweden. However, although only about 20% (*n* = 398) reported intake of folic acid fortified foods, those consuming such products had significantly higher folate intakes (296 µg/day, *Q*_1_ = 243, *Q*_3_ = 355) and erythrocyte folate concentrations (*n* = 48, 480 nmol/L, *Q*_1_ = 420, *Q*_3_ = 600) but not plasma folate concentrations (*n* = 51, 14 nmol/L, *Q*_1_ = 11, *Q*_3_ = 23). Due to the low consumption frequency of folic acid supplements and limited number of fortified foods, evaluation of DFE intakes resulted in a bimodal distribution making DFEs less relevant for evaluation of intakes in the Swedish population.

### Folate status and food patterns

The EFSA Panel considered erythrocyte folate concentrations the most reliable biomarker of folate status [17]. This is the first study presenting national data on erythrocyte folate concentration in Sweden. Erythrocyte folate concentration (median 460 nmol/L) was low compared to other countries without mandatory folic acid fortification. For example, in Denmark median erythrocyte folate concentrations in pregnant women was 840 nmol/L (week 18, *n* = 404); however, one third of these women reported daily consumption of a supplement containing 100 µg folic acid [14]. In an Irish population not consuming folic acid fortified foods or supplements median erythrocyte folate concentration was 699 nmol/L (*n* = 200) [15]. On the other hand, one should be cautious comparing data on folate status since the analytical methods, sampling and storage procedures might affect the results. The microbiological assay, as used in the Irish study, has been reported to result in higher results than immunoassays. For example, in NHANES the previously used Bio-Rad radioassay yielded 29% lower results for serum folate concentrations [33] and 45% lower results for erythrocyte folate concentrations [34] than the microbiological assay.

Median plasma folate concentration of 14 nmol/L in this study was rather high compared to previously reported plasma folate concentrations in Sweden ranging from 7.6 nmol/L up to 14.4 nmol/L (Table 4). Using the same analytical laboratory, Northern Europe (Sweden and Denmark) was found having significantly lower median plasma folate concentrations (10.7 nmol/L) than Central (13.9 nmol/L) and Southern Europe (13.7 nmol/L) [35]. In that study, plasma from the Malmö Diet and Cancer cohort was used [29], which is substantially higher than, e.g. from Northern Sweden [8] (Table 4) indicating that the difference might be even larger.

The prevalence of lower blood folate concentrations in the present study (4% using erythrocyte folate <340 nmol/L and 21% using plasma folate concentrations <10 nmol/L) was nearly identical with the pre-fortification prevalence in the USA (erythrocyte folate 3.5% and serum folate 24%) [36]. Folic acid supplement intake in US pre-fortification was 34% compared to 1% reporting folic acid supplement intake in this study (Table 1) and between 1% [5] and up to 32% [37] in Swedish cohort studies.

Prevalence rate of neural tube defects (NTD) in Sweden decreased significantly from 0.1% in 1999 to 0.08% in 2013 [38]. This might partly be explained by less than 1% reporting consumption of folic acid supplements during early pregnancy in 1999 compared to 15% in 2012 [38]. Our study was not designed to evaluate folate status in respect of risk of NTD. However, folate status during the first weeks of pregnancy is crucial for prevention of NTDs; hence, folate status among women in reproductive age was evaluated separately.

None of the women in reproductive age in this study had an erythrocyte folate concentration as recommended by WHO [24] to achieve optimal prevention against neural tube defects (>906 nmol/L). Median erythrocyte folate concentrations among women aged 18–45 years were 580 nmol/L (*Q*_1_ = 470 nmol/L; *Q*_3_ = 710 nmol/L), which according to Daly et al. [25] is associated with a nearly 3 times higher risk of NTDs compared with a status above 906 nmol/L. In countries with mandatory fortification, e.g. Canada median erythrocyte folate concentrations in the first trimester was 1280 nmol/L and only 10% have suboptimal concentrations (<906 nmol/L) [39].

Our results indicate difficulties in achieving an optimal folate status against prevention of NTDs via ordinary foods. Food groups associated with a higher folate status were a higher consumption of vegetables, pulses and roots as well as cheese and alcoholic beverages and lower consumption of meat. Apart from cheese and alcoholic beverages this is in line with the current Swedish dietary guidelines for adults as well as pregnant women. A high alcohol consumption results in intestinal malabsorption of folate, reduced liver uptake and increased urinary folate excretion [40] and is usually negatively associated with folate status, e.g. Pfeiffer et al. [36]. Hence, the positive association between folate status and alcohol intake in this survey probably indicates a food pattern beneficial for folate status. For example, both alcohol and cheese intake positively correlated with vegetable intake (*R* = 0.08, *p* < 0.001 for alcohol and *R* = 0.16, *p* < 0.0001 for cheese).

In order to achieve an optimal status during the first trimester, folic acid supplements and/or fortified foods appear to be the main choice, even for women consuming a diet rich in fruit and vegetables. However, targeted campaigns focusing on supplement use are proven to be less effective. In a survey in Denmark (*n* = 462), only 10% of the women reported taking folic acid supplements prior to pregnancy, despite the fact that more than 80% reported knowledge about the Danish recommendation to consume 400 µg folic acid supplements daily periconceptional [41]. In Sweden, only 21% among well-educated pregnant women (58% with college or university degree) reported folic acid supplements prior to pregnancy, despite that three in four had a planned pregnancy [42]. Thus, dietary advice, focusing on the whole diet and certain food groups, is important to facilitate improved folate intake and status. Unpublished results from simulation studies show that a balanced diet based on the NNR and Swedish dietary guidelines may provide amounts of folate in line with RI for women in childbearing ages (Elisabet Amcoff, personal communication).

This study aimed to be representative for the Swedish population. Nonetheless, the participation rate was low for both folate intake estimations (36%) and folate status assessments (30%). Participants had a higher education level than the average population, and this might have resulted in slightly higher estimated folate intakes (Table 2), which should be considered when interpreting the results. Neither education level nor income had an effect on folate status in this study (Table 3). However, the low participation rate in folate status assessment could have introduced bias. The participation rate was particularly low among men aged 18–44 years (participation rate between 23 and 29%) and immigrants (participation rate 27%) making the results less representable for these groups. For immigrants in Sweden, data on folate intake and status are limited. In a case–control study, the prevalence of high folate concentrations (defined as plasma folate concentrations >14 nmol/L) was significantly higher among pregnant women in Sweden born in a non-Nordic country compared to Nordic countries [28].

## Conclusions

Prevalence of low erythrocyte (<317 nmol/L) and plasma (<6.8 nmol/L) folate concentrations were low in Sweden, and estimated median intakes are well above average requirement. However, to obtain a folate status optimal for prevention against neural tube defects, major dietary changes would be necessary, and thus, folic acid supplements are generally required prior to conception, even for those consuming more than 500 g of fruit and vegetables daily.

## References

[1] Czeizel AE, Dudas I (1992). Prevention of the first occurrence of neural-tube defects by periconceptional vitamin supplementation. N Engl J Med.

[2] MRC (1991). Prevention of neural tube defects: results of the Medical Research Council Vitamin Study. The lancet.

[3] Norden (2014). Nordic nutrition recommendations 2012: integrating nutrition and physical activity.

[4] Ericson U, Sonestedt E, Gullberg B, Olsson H, Wirfält E (2007). High folate intake is associated with lower breast cancer incidence in postmenopausal women in the Malmö Diet and Cancer cohort. Am J Clin Nutr.

[5] Larsson SC, Bergkvist L, Wolk A (2008). Folate intake and risk of breast cancer by Estrogen and progesterone receptor status in a Swedish Cohort. Cancer Epidemiol Biomark Prev.

[6] Becker W (1994). Kostvanor och näringsintag i Sverige 1989. Resultat från undersökningen Hushållens livsmedelsutgifter och kostvanor (HULK).

[7] Becker W, Pearson M (2002). Riksmaten 1997-98 Kostvanor och näringsintag i Sverige-Metod-och resultatrapport.

[8] Van Guelpen B (2006) Folate in cancer and cardiovascular disease: prospective studies from the population-based northern Sweden health and disease study. Dissertation, Umeå University

[9] Enghardt Barbieri H, Pearson M, Becker W (2006) Riksmaten–barn 2003 Livsmedels-och näringsintag bland barn i Sverige [Riksmaten-Children 2003. The food and nutritional intake among children in Sweden]. National Food Agency, Uppsala

[10] Konings EJ, Roomans HH, Dorant E, Goldbohm RA, Saris WH, van den Brandt PA (2001). Folate intake of the Dutch population according to newly established liquid chromatography data for foods. Am J Clin Nutr.

[11] Finglas PM, van den Berg H, de Froidmont-Görtz I (1996). Improvements in the determination of vitamins in foods: method intercomparison studies and preparation of certified reference materials (CRMs). Food Chem.

[12] Bates CJ, Prentice A, van der Pols JC, Walmsley C, Pentieva KD, Finch S, Smithers G, Clarke PC (1998) Estimation of the use of dietary supplements in the National Diet and Nutrition Survey: people aged 65 years and over. An observed paradox and a recommendation. Euro J Clin Nutr 52(12):917–923 10.1038/sj.ejcn.1600665 9881887

[13] Busby A, Abramsky L, Dolk H, Armstrong B (2005). Preventing neural tube defects in Europe: population based study. Br Med J.

[14] Milman N, Byg KE, Hvas AM, Bergholt T, Eriksen L (2006). Erythrocyte folate, plasma folate and plasma homocysteine during normal pregnancy and postpartum: a longitudinal study comprising 404 Danish women. Eur J Haematol.

[15] Hopkins SM, Gibney MJ, Nugent AP, McNulty H, Molloy AM, Scott JM, Flynn A, Strain JJ, Ward M, Walton J, McNulty BA (2015). Impact of voluntary fortification and supplement use on dietary intakes and biomarker status of folate and vitamin B-12 in Irish adults. Am J Clin Nutr.

[16] Pounis G, Di Castelnuovo AF, de Lorgeril M, Krogh V, Siani A, Arnout J, Cappuccio FP, van Dongen M, Zappacosta B, Donati MB (2014). Folate intake and folate serum levels in men and women from two European populations: The IMMIDIET project. Nutrition.

[17] EFSA (2014). Scientific opinion on dietary reference values for folate. EFSA J.

[18] Amcoff E, Edberg A, Enghardt Barbieri H, Lindroos A, Nälsén C, Pearson M, Warensjö Lemming E (2012) Riksmaten–vuxna 2010–11. Livsmedels-och näringsintag bland vuxna i Sverige (Dietary habits and nutrient intake in Sweden 2010–11. The third national food consumption survey). National Food Agency, Uppsala

[19] Nybacka S, Forslund HB, Wirfält E, Larsson I, Ericson U, Warensjö Lemming EW, Bergström G, Hedblad B, Winkvist A, Lindroos AK (2016). Comparison of a web-based food record tool and a food frequency questionnaire and objective validation using the doubly labeled water technique in a Swedish middle-aged population. J Nutr Sci.

[20] Nybacka S, Lindroos AK, Wirfält E, Leanderson P, Landberg R, Ericson U, Larsson I, Warensjö Lemming E, Bergström G, Hedblad B, Orho-Melander M, Melander O, Winkvist A, Bertéus Forslund H (2016). Carotenoids and alkylresorcinols as objective biomarkers of diet quality when assessing the validity of a web-based food record tool and a food frequency questionnaire in a middle-aged population. BMC Nutr.

[21] Lemming EW, Nälsén C, Becker W, Ridefelt P, Mattisson I, Lindroos AK (2015). Relative validation of the dietary intake of fatty acids among adults in the Swedish National Dietary Survey using plasma phospholipid fatty acid composition. J Nutr Sci.

[22] Öhrvik V, Carlsen MH, Källman A, Martinsen TA (2015). Improving food composition data by standardizing calculation methods. Tema nord.

[23] Suitor C, Bailey L (2000). Dietary folate equivalents: interpretation and application. J Am Diet Assoc.

[24] WHO (2015). Guideline: optimal serum and red blood cell folate concentrations in women of reproductive age for prevention of neural tube defects.

[25] Daly LE, Kirke PN, Molloy A, Weir DG, Scott JM (1995). Folate levels and neural tube defects: implications for prevention. J Am Med Assoc.

[26] Ruiz JR, Hurtig-Wennlöf A, Ortega FB, Patterson E, Nilsson TK, Castillo MJ, Sjöström M (2007). Homocysteine levels in children and adolescents are associated with the methylenetetrahydrofolate reductase 677C>T genotype, but not with physical activity, fitness or fatness: The European Youth Heart Study. Br J Nutr.

[27] Lundqvist A, Johansson I, Wennberg A, Hultdin J, Hogberg U, Hamberg K, Sandstrom H (2014). Reported dietary intake in early pregnant compared to non-pregnant women a cross-sectional study. BMC Pregnancy Childbirth.

[28] George L, Mills JL, Johansson AV (2002). Plasma folate levels and risk of spontaneous abortion. J Am Med Assoc.

[29] Ericson UC, Ivarsson MI, Sonestedt E, Gullberg B, Carlson J, Olsson H, Wirfält E (2009). Increased breast cancer risk at high plasma folate concentrations among women with the MTHFR 677T allele. Am J Clin Nutr.

[30] Wahlin A, Backman L, Hultdin J, Adolfsson R, Nilsson LG (2002). Reference values for serum levels of vitamin B12 and folic acid in a population-based sample of adults between 35 and 80 years of age. Public Health Nutr.

[31] Björkegren K, Svärdsudd K (2003). Reported symptoms and clinical findings in relation to serum cobalamin, folate, methylmalonic acid and total homocysteine among elderly Swedes: a population-based study. J Intern Med.

[32] The Swedish Food Composition Database (2016) National Food Agency. http://www.slv.se/en-gb/Group1/Food-and-Nutrition/The-Food-Database/. Accessed 09 Feb 2016

[33] Fazili Z, Pfeiffer CM, Zhang M (2007). Comparison of serum folate species analyzed by LC-MS/MS with total folate measured by microbiologic assay and bio-rad radioassay. Clin Chem.

[34] Pfeiffer CM, Hughes JP, Durazo-Arvizu RA, Lacher DA, Sempos CT, Zhang M, Yetley EA, Johnson CL (2012). Changes in measurement procedure from a radioassay to a microbiologic assay necessitate adjustment of serum and RBC folate concentrations in the U.S. Population from the NHANES 1988–2010. J Nutr.

[35] Eussen SJ, Nilsen RM, Midttun Ø, Hustad S, Jssennagger IN, Meyer K, Fredriksen Å, Ulvik A, Ueland PM, Brennan P (2013). North–south gradients in plasma concentrations of B-vitamins and other components of one-carbon metabolism in Western Europe: results from the European Prospective Investigation into Cancer and Nutrition (EPIC) Study. Br J Nutr.

[36] Pfeiffer CM, Sternberg MR, Fazili Z, Lacher DA, Zhang M, Johnson CL, Hamner HC, Bailey RL, Rader JI, Yamini S, Berry RJ, Yetley EA (2015). Folate status and concentrations of serum folate forms in the US population: National Health and Nutrition Examination Survey 2011–2. Br J Nutr.

[37] Ericson U, Borgquist S, Ivarsson MI, Sonestedt E, Gullberg B, Carlson J, Olsson H, Jirström K, Wirfält E (2010). Plasma folate concentrations are positively associated with risk of estrogen receptor β negative breast cancer in a Swedish nested case control study. J Nutr.

[38] Socialstyrelsen (2015). Birth defects 2013.

[39] Shrim A, Kapur B, Snyder J, Basso O, Blank D, Brown RN (2015). Maternal predictors of RBC folate levels in an urban Canadian population. Reprod Toxicol.

[40] Medici V, Halsted CH (2013). Folate, alcohol, and liver disease. Mol Nutr Food Res.

[41] Friberg AK, Jorgensen FS (2015). Few Danish pregnant women follow guidelines on periconceptional use of folic acid. Danish Med J.

[42] Tyden T, Stern J, Nydahl M, Berglund A, Larsson M, Rosenblad A, Aarts C (2011). Pregnancy planning in Sweden—a pilot study among 270 women attending antenatal clinics. Acta Obstet Gynecol Scand.

